# Knowledge, attitudes and practices related to tuberculosis in pharmacy workers in a cross-sectional survey in El Agustino, Peru

**DOI:** 10.1371/journal.pone.0196648

**Published:** 2018-07-24

**Authors:** Patricia J. García, Gustavo Hernández-Córdova, Paria Pourjavaheri, Hilbert J. Gómez-Paredes, Samuel Sudar, Angela M. Bayer

**Affiliations:** 1 Epidemiology, STI, and HIV Research Unit, School of Public Health and Administration, Universidad Peruana Cayetano Heredia, Lima, Peru; 2 David Geffen School of Medicine, University of California Los Angeles, Los Angeles, California, United States of America; Asociacion Civil Impacta Salud y Educacion, PERU

## Abstract

**Introduction:**

Although the worldwide incidence of tuberculosis (TB) is slowly decreasing, annual infection rates in Peru remain among the highest in the Americas. Pharmacies could play an important role in facilitating early detection of TB. However, the awareness, expertise and cooperation of pharmacy workers is fundamental. This study explored the TB-related knowledge, attitudes and practices of pharmacy workers in a district with one of the highest incidences of TB in Peru.

**Methods:**

This cross-sectional study applied a questionnaire that was administered face to face using smartphones with one pharmacy worker at each of 45 randomly selected pharmacies in the El Agustino district of Lima, Peru.

**Results:**

Participants were primarily female (78%) and had an average age of 31.3 years old (range 18–57 years old). Only 11% of participants were pharmacists with complete university training. The pharmacy workers’ knowledge was adequate; however, workers had important knowledge gaps and myths regarding prevention of TB transmission. Most pharmacy workers (77%) reported they would send a client with a history of cough for more than two weeks to a healthcare center, while 23% reported they would offer them antitussive medication or antibiotics. Almost all workers reported talking with clients about diseases and reported respiratory symptoms as one of the most common causes for consultation (60%). Most participants expressed interest in learning more about TB and expanding their involvement in the fight against TB in their community.

**Conclusion:**

Pharmacy workers have adequate knowledge about TB. However, we identified gaps in knowledge with respect to prevention of TB transmission. Pharmacy workers commonly see patients with respiratory symptoms and some offer recommendations, including for treatment. Pharmacy workers are willing to learn more and contribute to TB control and could be a valuable asset in the control and prevention of TB in Peru. To achieve this integration of pharmacy workers into TB control and prevention, more research is needed.

## Introduction

According to the World Health Organization (WHO), tuberculosis (TB) affects 117 per 100,000 inhabitants of Peru, one of the highest TB incidence rates in the America [1]. Evidence shows that TB incidence is positively correlated with high social inequality and incidence is about 4–5 times greater in countries with lower health expenditure per capita [2]. Resistance to anti-tuberculosis drugs is an expanding problem that has complicated the control of TB in Peru, where there has been an increase in cases of multi-drug resistant TB (MDR-TB) and extensively drug-resistant TB (XDR-TB) [3]. Peru is on the list of the top 10 countries with the highest MDR-TB incidence rate globally [1]. Between 2004 and 2011 the Peruvian government increased its budget towards fighting TB by 600% [4]. In 2016, a new law, Law 30287: Law for the Control and Prevention of Tuberculosis in Peru, declared the country’s fight against TB as an issue of national interest [5]. Despite these efforts, and although Peru’s Human Development Index has increased, TB-related mortality remains as high as 6.3 cases per 100,000 population in Peru, compared with 2.3 cases per 100,000 reported for the region of the Americas [1,6].

Primary healthcare services, which are key to TB diagnosis and control, are also overburdened globally and in Peru. According to the WHO, “primary care brings promotion and prevention, cure and care together in a safe, effective and socially productive way at the interface between the population and the health system” [7]. However, the demands on primary healthcare services are growing worldwide. Under the national guidelines developed and approved by the Peruvian National Health Strategy for TB Prevention and Control, TB screening takes place predominately at primary healthcare centers [8]. Among all people attending health centers, healthcare providers identify patients who have had a cough for more than 2 weeks (“sintomático respiratorio or SR” in Spanish, meaning “with respiratory symptoms”) and request two consecutive sputum samples for an Acid-fast bacilli (AFB) smear. If at least one sputum smear is positive, it is considered a TB case [8–9].

Globally and in Peru, many health interventions aimed at reaching underserved populations have used pharmacies as the first point of contact for potential patients [10–11] and proven pharmacies to be potential partners in combatting TB [12–13]. Pharmacies could represent a key venue for complementing current TB prevention and control efforts in Peru. Therefore, the objective of this study was to assess the TB-related knowledge, attitudes and practices of pharmacists and pharmacy workers in the district of El Agustino in Lima, Peru using smartphone-based questionnaires.

## Methods

### Ethics statement

This project was approved by the Institutional Review Boards of the Universidad Peruana Cayetano Heredia (#6355) and the Health Directorate for the Lima East Region—Lima IV. All participants were 18 years of age or older and provided verbal informed consent prior to initiating participation. Participants did not receive any monetary incentives.

### Study setting

We implemented this cross sectional study in the El Agustino district of Peru’s capital city of Lima. El Agustino has an estimated population of 191,365 inhabitants and is under the jurisdiction of the Health Directorate for the Lima East Region (DISA-Lima IV). The Ministry of Health (MOH) is the largest provider of health services in Peru. El Agustino has 10 MOH health establishments, including one general hospital, eight health centers, and one health post. MOH outpatient services are open from 8 am to 2 pm and are always busy. The hospital provides emergency services 24 hours a day and health centers provide urgent services that are open only until 8 pm [14]. El Agustino was chosen for this study due to its high TB prevalence and incidence. In 2013, the TB prevalence in El Agustino was 272.3 per 100,000 inhabitants and the incidence was 140.2 per 100,000 inhabitants [15].

### Study participants

In Peru, Law 29459 regulates all aspects of pharmaceutical products, medical devices, and health products. Formal drug-dispensing establishments, including pharmacies and ‘boticas,’ are registered with DEMID (Dirección Ejecutiva De Medicamentos, Insumos Y Drogas), an office of DIGEMID (Dirección General de Medicamentos, Insumos y Drogas), Peru’s Drug Regulatory Authority [16]. A list of the 109 pharmacies and boticas in El Agustino was obtained from DEMID. Both pharmacies and ‘boticas’ are allowed to sell and dispense medications. The difference between the two types of establishments is their ownership and management: while pharmacies are managed by pharmacists, ‘boticas’ are not managed by pharmacists. Both types of businesses are referred to as ‘pharmacies’ throughout this paper [11].

We estimated a sample size of 51 pharmacies for the study (including a 10% refusal to participate) and used simple random sampling to select the participating pharmacies. We created a list of all of the pharmacies in the district and used a random numbers table to select the 51 that were invited to participate. If a pharmacy was found to be closed, it was revisited twice and if it was still closed, it was replaced with another pharmacy using the same method. We only replaced pharmacies once. At each pharmacy, the interviewer explained the study to the person in charge and invited the pharmacy worker with the following criteria to participate: the persona who had been working at the establishment for the longest period of time and who was working during the study team’s visit. The survey was initiated after obtaining informed consent.

### Data collection and data analysis

The data collection instrument was adapted from previously developed tools used in Peru or other settings heavily impacted by TB [17–18]. The survey was divided into the following sections: pharmacy characteristics, participant demographics and general practices at the pharmacy; TB-related knowledge; TB-related attitudes; TB-related practices; interest in receiving more information about TB; and the availability and use of the Internet and mobile phones. The survey was piloted in an area of Lima that is similar to El Agustino (outlying district, high TB incidence and prevalence). The survey was applied by an interviewer using a cellphone programmed with Magpi (Magpi, Washington, D.C, USA) and lasted around 20 minutes.

Descriptive statistics were calculated for all the survey questions. Frequencies and percentages were calculated using Microsoft Excel version 14.6.8 (Microsoft Corporation, CA, USA).

## Results

From the 51 randomly selected pharmacies, five were closed and replaced as explained in the Methods section. Six pharmacies declined participation. This analysis includes interviews with the 45 pharmacy workers who agreed to participate.

### Pharmacies’ characteristics

The great majority of participating pharmacies were boticas (87%) and almost one-third (31%) were a part of a franchise, which means that they had more than one location in Peru. Pharmacies opened for an average of 82.7 hours per week, although with a wide range of 11.5–112.0 hours per week. Most pharmacies worked from 7 am to 11 pm. On average, pharmacies had a total of 3.8 workers (range 1–10), with 2.6 workers per shift. Based on the reported number of shifts, workers and average number of clients per shift, we estimate that each pharmacy sees an average of 363.3 clients per day.

### Participants’ demographic characteristics

Of the 45 participants, 35 (77.8%) were women and their average age was 31.4 years (range 18–57). Most participants (86.7% or 39/45) were pharmacy technicians, which means that they completed three years of technical school training, and very few (11.1% or 5/45) were pharmacists, with a university degree in pharmacy. Few participants (17.8% or 8/45) were pharmacy owners. Participants’ average time working at pharmacies was 5.7 years (range 3 months-10 years), although almost one-third (31.1% or 14/35) had less than one year of work experience in this field (Table 1).

**Table 1:** Participants’ demographic characteristics, pharmacy workers, El Agustino, Lima, Peru (N = 45).

Characteristics	N (%)
**Gender**	
Female	35 (77.8)
**Age**	
18–25	20 (44.4)
26–35	11 (24.4)
36+	12 (26.7)
Unknown	2 (4.5)
**Occupation**	
Pharmacist	5 (11.1)
Pharmacy Technician	39 (86.7)
Other	1 (2.2)
**Years of Experience**	
Less than 1 year	14 (31.1)
1–5 years	19 (42.3)
6–10 years	6 (13.3)
Greater than 10 years	6 (13.3)

Nearly all participants (93.3% or 42/45) had received some form of training prior to commencing work at the pharmacy. Most participants (75.6% or 34/45) reported receiving additional training about various health topics within the last 12 months: 18/34 (52.9%), from the employer; 14/34 (41.2%) from a pharmaceutical company; and 10/34 (29.4%) from the Ministry of Health. The topics of the training were pharmaceutical products (79.4% or 27/34), recognition of common diseases (35.3% or 12/34); and management of common diseases (20.6% or 7/34).

### Participants’ TB-related knowledge and attitudes

All participants acknowledged having heard about TB. Interestingly, one-third of participants (35.6% or 16/45) reported having had a relative or close friend with TB. When asked about how much they felt that they know about TB: 19/45 (42.2%) considered themselves poorly informed; 18/45 (40.0%) felt reasonably informed; and only 7/45 (15.6%) felt very well-informed. Table 2 presents the percentage of participants with correct answers on each of the knowledge-related topics evaluated. Almost all participants (95.6% or 43/45) correctly recognized both that TB is frequent in Peru and that drug-resistant TB is a serious problem in the country. However, fewer participants (71.1% or 32/45) were aware that TB is very common in El Agustino. Participants were almost universally aware (97.8% or 44/45) that TB transmission is airborne. However, only one-quarter of participants (24.4% or 11/45) correctly identified effective ways to avoid TB transmission, such as covering one’s mouth when coughing and opening the windows while travelling on buses. A significant proportion of participants (42.2% or 19/45) incorrectly identified sharing eating utensils as a means of TB transmission, affirming the persistence of common myths. Around eight in ten participants knew that TB is curable, could identify sputum evaluation as an effective diagnostic test for TB, knew that TB treatment regimens given by public health establishments are the most effective, and could identify cough as the most common TB symptom. Around seven in ten participants correctly recognized the consequences of not completing TB treatment or taking the medication irregularly and acknowledged that TB is highly contagious. Finally, and importantly, the great majority of participants (88.9% or 40/45) indicated that they would be interested in learning more about TB and participating in prevention activities in their community. They suggested having workshops (55.6% or 25/45), internet courses (51.1% or 23/45), brochures (24.4% or 11/45) and the use of cellular phone information messages (17.8% or 8/45).

**Table 2:** Participants’ TB-related knowledge as demonstrated by the percentage of correct answers per question, pharmacy workers, El Agustino, Lima, Peru (N = 45).

Question/Topic	n	%
Knowledge that TB transmission is airborne	44	97.8
Knowledge that TB is frequent in Peru	43	95.6
Knowledge that drug-resistant TB is a serious problem in Peru	43	95.6
Knowledge that TB is almost always a curable disease	37	82.2
Knowledge that sputum evaluation is an effective diagnostic test for pulmonary TB	37	82.2
Knowledge that the most effective TB treatment is given by public health establishments	36	80.0
Knowledge that cough is the most common symptom of pulmonary TB	35	77.8
Knowledge that TB is frequent in El Agustino	32	71.1
Knowledge of the consequences of not completing TB treatment or taking it irregularly	32	71.1
Knowledge that TB is highly contagious	30	66.7
Knowledge of ways to avoid transmission of TB	11	24.4

Over half of participants (55.6% or 25/45) reported that they very much like to talk with their clients about health complaints and problems and/or diseases when they come to the pharmacy and almost half of participants (44.4% or 20/45) reported that they also like to talk with their clients about treatment options. When asked about how they would feel talking about TB with their clients, the great majority (84.4% or 38/45) responded that they would feel comfortable or very comfortable.

### Participants’ practices with pharmacy clients

Most participants (91.1% or 41/45) stated that they talk with clients about their health problems (for an average of 5.8 times per day, range 1–30) and that they provide treatment recommendations (for an average of 6.2 times per day, range 1–40). At the same time, however, most participants reported that the main reason that they did not engage more in these activities was lack of time. When asked about the most common health problems presented by clients at pharmacies, the most common by far were respiratory problems (60.0% or 27/45), followed by gastrointestinal problems (26.7% or 12/45).

We also asked participants what they usually do when a client presents with a cough that has lasted for more than 2 weeks. The majority (75.6% or 34/45) reported they would tell the client to go to a health center. However, 22.2% (10/45) answered that they were more likely to offer them either antitussive medication (17.7% or 8/45) or antibiotics (4.5% or 2/45) instead. Interestingly, one person did not want to answer this question.

## Discussion

This study shows that almost all pharmacy workers have heard about TB, more than a third report a close contact or a relative with TB, and most have good TB-related knowledge. However, they have gaps in knowledge related to how to avoid transmission of TB and continue to believe myths regarding disease transmission. Pharmacy workers see an important number of clients each day. Almost all workers reported talking with clients about health complaints, with respiratory symptoms representing the most common complaint. Three in four workers said that they would tell a client with a persistent cough to go to a health center, although almost one in five pharmacy workers reported recommending antitussives or antibiotics to these clients. Importantly, most pharmacy workers expressed interest in learning more about TB and in expanding their involvement in the fight against TB in their community.

The characteristics of the pharmacy workers in Peru have been described in earlier studies of pharmacies and sexually transmitted infections (STIs) [11] and are similar to our findings. As in other low- and middle-income countries (LMICs), pharmacy workers are in contact with an important number of clients and respond to their needs for counseling and even treatment for their health complaints [19]. The great majority of participants in the current study stated that they provide treatment recommendations to their clients. Despite efforts in regulation, in LMICs, many prescription medications are available without a prescription. Although those practices may not be “legal,” they fill a gap that exists in the formal health system, offering clients accessibility (“closer to home”), acceptability (pharmacy workers are perceived by clients as knowledgeable and non-judgmental), and affordability (no consultation fee) [20–21]. A previous study with Tanzanian pharmacy workers suggested that financial gain was a motivating factor for providing medications to clients [22]. Pharmacies are businesses and they are always looking for opportunities to sell medications. The profit margin varies according to the product and some laboratories have mechanisms to incentivize workers to sell their products such as raffles of airplanes tickets. However pharmacy workers also believe that they fulfill a role in their community and they want to do good [11].

A study in Syria found that the sale of antibiotics without a prescription was common practice despite federal guidelines [23]. Such patterns have also been found for TB treatment in other settings where health providers’ knowledge about TB was low [24]. In the case of TB, it would be important to better understand incentives that could impact an intervention in Peru.

A scoping review of knowledge, attitudes and practices of private sector providers of TB care found that all categories of TB health workers lacked extensive knowledge of national treatment guidelines. Moreover, there were no systematic procedures implemented for referrals, record keeping, treatment monitoring or case holding [25]. Past studies in other contexts have shown that communication strategies, educational campaigns, training and interventions, and regulatory enforcement have the potential to improve pharmacy workers’ attitudes and practices towards the correct dispensation of drugs and are essential in improving compliance with national guidelines and regulations [26–28].

In our study context, an area with high TB incidence, one of the most common complaints reported at pharmacies were respiratory symptoms. Studies in other settings have shown that for at least 42% of patients with respiratory symptoms who were later diagnosed with TB, their first care-seeking interaction was at pharmacies [29–30]. Pharmacies have also been reported as the first point of contact for most TB patients, especially those in urban settings. For example in Delhi, India, pharmacy workers were the first point of contact and clinical advice for 2/3 of TB patients [31]. Unfortunately, studies that backtrack to identify and record the pathways followed by TB patients showed that almost none of the drug sellers referred probable TB patients directly to a health center, causing delays in the diagnosis and treatment of TB [21,31].

On the other hand, a study of diagnostic delays and associated factors among patients with pulmonary TB in Tanzania found that patients living far from pharmacies were less likely to visit a health center. This may be related to recommendations given by pharmacies to seek treatment at a health center or to the availability of health services, which affects use by residents [32]. In our study, three out of four pharmacy workers said that they refer cases with cough for more than two weeks to a health center. However, there could be social desirability bias in their reporting. Nevertheless, one key concern is that one in five of the pharmacy workers reported they treat those cases with cough medications and even with antibiotics, without referring them to health providers who would carry out diagnostic testing and provide appropriate treatment. This is an issue that needs to be addressed. Although cough is a very common symptom observed in different diseases, including many minor illnesses [33], it is a very frequent symptom of TB that is of prime importance for transmitting the infection [34]. In high incidence areas such as our study context, it is of critical importance to use the case detection definition of “cough lasting more than 2 weeks (or between 2 to 3 weeks),” as the WHO, the International Union Against Tuberculosis and Lung Disease, and the Royal Netherlands Tuberculosis Association recommend [9]. Given the high number of clients with respiratory symptoms who come to pharmacies as their first point of care, this may represent an opportunity to catch TB cases early and reduce the delays in diagnosis and initiation of treatment. It would be important to test this intervention to evaluate its feasibility, acceptability and effectiveness.

In Bolivia, an intervention to promote referrals from private pharmacies to the National Tuberculosis Program (NTBP) found a decrease in the proportion of pharmacies selling TB medication and an increase in the percentage of pharmacies that referred simulated clients seeking TB drugs to the NTBP, with patient referrals increasing from 22% to 58% after the intervention [13]. In Cambodia, pharmacies collaborating with the country’s NTBP have referred symptomatic respiratory individuals to public health centers for treatment since 2005. The Phnom Penh Municipal Health Department estimates that pharmacy-referred patients account for approximately 9% of all TB patients in Cambodia [24]. A recent systematic review on the role of community pharmacists for the screening and management of poorly controlled asthma and chronic obstructive pulmonary disease (COPD) delineated potential roles of pharmacists in primary care: timely screening, referral of at-risk individuals to primary healthcare providers, and ongoing support and management for chronic conditions [19]. When provided adequate training, pharmacists and pharmacy workers in different settings have been shown to be valuable partners in the detection of a number of conditions including poorly controlled asthma and COPD [19] anemia, diabetes and hypertension [35–36]. A previous study in Peru implemented a network of trained pharmacy workers and physicians that was acceptable at the community level and was effective for the prevention and management of STIs [37–38].

A limitation of our study is that it was carried out only in one district and may not represent what happens in the rest of Lima or Peru. Additionally, we collected information from only one of the workers at each pharmacy, who reported what he/she thought happened during other shifts and estimated the numbers of clients coming to the pharmacy. Finally, the study was not powered to analyze associations. An important strength of this study is that it is the first one in Peru to explore pharmacy workers’ TB-related knowledge, attitudes and practices while at work. Additionally, the study included around 40% of all pharmacies in a district with one of the highest rates of TB. Finally, we used cell phones to collect the data, taking less participant time and decreasing inconsistencies in the data.

Training, supervision and involvement of pharmacy workers in promoting TB screening, creating new points for collection of sputum samples at pharmacies (which are much closer to patients than health services and open for longer hours) and assuring recording and reporting of the potential cases seen at pharmacies, may help to reduce TB transmission, decrease the number of cases and improve the outcomes of patients with TB.

## Conclusions

In conclusion, we found that pharmacy workers have adequate knowledge about TB, but with gaps and myths related to ways to prevent transmission. They commonly see clients with respiratory symptoms and some workers are incorrectly prescribing antibiotics to individuals that meet the case definition for a person with TB, instead of promoting screening for TB or referring to a health center. Most participants are interested in learning more about TB, report feeling comfortable with the possibility of talking about TB with clients, and would like to get involved in actions to fight TB in their community. Pharmacy workers may be a valuable asset in the control and prevention of TB in Peru. However, to support the implementation of an intervention related to TB at pharmacies, future research is needed on how to train and involve pharmacy workers in the promotion of TB screening, the promotion of health seeking behaviors by referring and encouraging people with respiratory symptoms to go to health centers, the reporting of possible TB cases, and the use of technologies to assure follow up of clients. We need to decrease delays in TB diagnosis and include pharmacies as a key strategy and additional tool in the control of TB in high burden countries like Peru.

## Supporting information

S1 Database Database with survey results. (XLS)

S1 File Data collection instrument. Survey used to collect data from participants.
(PDF)

## References

[1] World Health Organization (WHO). Global Tuberculosis Report 2017. Geneva: WHO; 2017. http://www.who.int/tb/publications/global_report/en/. Last accessed 1-30-2018

[2] Munayco CV, Mujica OJ, Leon FX, del Granado M, Espinal MA. Social determinants and inequalities in tuberculosis incidence in Latin America and the Caribbean. Rev Panam Salud Publica. 2015;38(3):177–85. 26757995

[3] Alarcon V, Alarcon E, Figueroa C, Mendoza-Ticona A. [Tuberculosis in Peru: epidemiological situation, progress and challenges for its control]. Rev Peru Med Exp Salud Publica. 2017;34(2):299–310 29177392 10.17843/rpmesp.2017.342.2384

[4] Ministerio de Salud del Perú (MINSA). [The current situation with tuberculosis in Peru]. Situación de la tuberculosis en el Perú. Lima, Perú: MINSA, 2011. https://www.minsa.gob.pe/portada/Especiales/2011/respiravida/archivos/Ayuda_memoria_Lanzamiento_TB.pdf. Last accessed 1-30-2018

[5] Congreso de la República. [Law to Prevent and Control Tuberculosis in Peru]. Ley N° 30287 Ley de Prevención y Control de la Tuberculosis en el Perú. 2015. http://www.leyes.congreso.gob.pe/Documentos/Leyes/30287.pdf. Last accessed 1-30-2018

[6] Castaneda-Hernandez DM, Tobon-Garcia D, Rodriguez-Morales AJ. [Association between tuberculosis incidence and the Human Development Index in 165 countries of the world]. Rev Peru Med Exp Salud Publica. 2013;30(4):560–8. 24448930

[7] World Health Organization (WHO). The World Health Report 2008: Primary Health Care—Now More Than Ever. Geneva: WHO; 2008.

[8] Ministerio de Salud del Perú (MINSA). [Technical guideline for tuberculosis control in Peru.] Norma técnica para el control de la tuberculosis en el Perú. Lima, Perú: MINSA; 2013. ftp://ftp2.minsa.gob.pe/normaslegales/2013/RM715_2013_MINSA.pdf. Last accessed 1-30-2018

[9] World Health Organization, International Union Against Tuberculosis and Lung Disease, Royal Netherlands Tuberculosis Association. Revised international definitions in tuberculosis control. Int J Tuberc Lung Dis 2001; 5: 213–5. 11326818

[10] McGuire TR, Leypoldt M, Narducci WA, Ward K. Accessing rural populations: role of the community pharmacist in a breast and cervical cancer screening programme. J Eval Clin Pract. 2007;13(1):146–9. 10.1111/j.1365-2753.2007.00677.x 17286737

[11] Garcia PJ, Gotuzzo E, Hughes JP, Holmes KK. Syndromic management of STDs in pharmacies: evaluation and randomised intervention trial. Sex Transm Infect. 1998;74 Suppl 1:S153–8. 10023367

[12] Jakeman B, Gross B, Fortune D, Babb S, Tinker D, Bachyrycz A. Evaluation of a pharmacist-performed tuberculosis testing initiative in New Mexico. J Am Pharm Assoc (2003). 2015;55(3):307–12. 26003159 10.1331/JAPhA.2015.14141

[13] Lambert ML, Delgado R, Michaux G, Vols A, Speybroeck N, Van der Stuyft P. Collaboration between private pharmacies and national tuberculosis programme: an intervention in Bolivia. Trop Med Int Health. 2005;10(3):246–50. 10.1111/j.1365-3156.2004.01383.x 15730509

[14] Data on Peruvian health establishments. http://www.geominsa.minsa.gob.pe:8080/geominsa/. Last accessed 1-30-2018

[15] Ministerio de Salud (MINSA). [Health Situation Analysis. IV Lima East Health Directorate.] Análisis de Situación de Salud. DISA IV Lima Este. Lima, Perú: MINSA; 2014. http://bvs.minsa.gob.pe/local/MINSA/3326.pdf. Last accessed 1-30-2018

[16] Dongo V. [Law 29459. Law on pharmaceutical products, medical devices and health products.] Rev Peru Med Exp Salud Publica. 2009;26(4):517–529.

[17] Islam QS, Ahmed SM, Islam MA, Chowdhury AS, Siddiquea BN, Husain MA. Informal allopathic provider knowledge and practice regarding control and prevention of TB in rural Bangladesh. Int Health. 2014;6(3):225–31. 10.1093/inthealth/ihu025 24938278

[18] Kiefer EM, Shao T, Carrasquillo O, Nabeta P, Seas C. Knowledge and attitudes of tuberculosis management in San Juan de Lurigancho district of Lima, Peru. J Infect Dev Ctries. 2009;3(10):783–8. 20009280 10.3855/jidc.335

[19] Fathima M, Naik-Panvelkar P, Saini B, Armour CL. The role of community pharmacists in screening and subsequent management of chronic respiratory diseases: a systematic review. Pharm Pract (Granada). 2013;11(4):228–45. 24367463 10.4321/s1886-36552013000400008 PMC3869639

[20] Garcia PJ, Carcamo CP, Chiappe M, Holmes KK. Sexually transmitted and reproductive tract infections in symptomatic clients of pharmacies in Lima, Peru. Sex Transm Infect. 2007;83(2):142–6. 10.1136/sti.2006.022657 16916881 PMC2598629

[21] Sarker M, Mohammad D, Paul S, Akter R, Islam S, Biswas G, et al Lost in care pathway: a qualitative investigation on the health system delay of extra pulmonary tuberculosis patients in Bangladesh.BMC Health Serv Res. 2017;17(1):240 10.1186/s12913-017-2181-8 28351361 PMC5370471

[22] Larsson M, Odberg Pettersson K, Kashiha J, Ross MW, Agardh A. Stretching the Boundaries: Tanzanian Pharmacy Workers’ Views and Experiences of Providing STI Services for Men Who Have Sex with Men. PLoS One. 2016;11(11):e0166019 10.1371/journal.pone.0166019 27812206 PMC5094583

[23] Al-Faham Z, Habboub G, Takriti F. The sale of antibiotics without prescription in pharmacies in Damascus, Syria. J Infect Dev Ctries. 2011;5(5):396–9 21628818 10.3855/jidc.1248

[24] Bell CA, Eang MT, Dareth M, Rothmony E, Duncan GJ, Saini B. Provider perceptions of pharmacy-initiated tuberculosis referral services in Cambodia, 2005–2010. Int J Tuberc Lung Dis. 2012;16(8):1086–91. 10.5588/ijtld.11.0669 22687275

[25] Bell CA, Duncan G, Saini B. Knowledge, attitudes and practices of private sector providers of TB care: a scoping review. Int J Tuberc Lung Dis. 2011;15(8):1005–17. 10.5588/ijtld.10.0294 21669027

[26] Philip S, Isaakidis P, Sagili KD, Meharunnisa A, Mrithyunjayan S, Kumar AM. "They know, they agree, but they don’t do"—the paradox of tuberculosis case notification by private practitioners in Alappuzha district, Kerala, India. PLoS One. 2015;10(4):e0123286 10.1371/journal.pone.0123286 25909330 PMC4409371

[27] Soumya R, Devarashetty V, Jayanthi CR, Sushma M. Drug dispensing practices at pharmacies in Bengaluru: A cross-sectional study. Indian J Pharmacol. 2016;48(4):360–4. 10.4103/0253-7613.186204 27756944 PMC4980921

[28] Pham DM, Byrkit M, Pham HV, Pham T, Nguyen CT. Improving pharmacy staff knowledge and practice on childhood diarrhea management in Vietnam: are educational interventions effective? PLoS One. 2013;8(10):e74882 10.1371/journal.pone.0074882 24098355 PMC3789740

[29] Storla DG, Yimer S, Bjune GA. A systematic review of delay in the diagnosis and treatment of tuberculosis.BMC Public Health. 2008;8:15 10.1186/1471-2458-8-15 18194573 PMC2265684

[30] Steen TW, Mazonde GN.Pulmonary tuberculosis in Kweneng District, Botswana: delays in diagnosis in 212 smear-positive patients.Int J Tuberc Lung Dis. 1998;2(8):627–34. 9712276

[31] Kapoor SK, Raman AV, Sachdeva KS, Satyanarayana S. How did the TB patients reach DOTS services in Delhi? A study of patient treatment seeking behavior. Neyrolles O, editor. PLoS ONE. 2012;7:e42458 10.1371/journal.pone.0042458 22879990 PMC3412865

[32] Said K, Hella J, Mhalu G, Chiryankubi M, Masika E, Maroa T, et al Diagnostic delay and associated factors among patients with pulmonary tuberculosis in Dar es Salaam, Tanzania.Infect Dis Poverty. 2017;6(1):64 10.1186/s40249-017-0276-4 28335816 PMC5364704

[33] De Blasio F, Virchow JC, Polverino M, Zanasi A, Behrakis PK, Kilinc G, et al Cough management: a practical approach. Cough. 2011;7(1):7 10.1186/1745-9974-7-7 21985340 PMC3205006

[34] Turner RD, Bothamley GH. Cough and the transmission of tuberculosis.J Infect Dis. 2015;211(9):1367–72. 10.1093/infdis/jiu625 25387581

[35] Saldarriaga EM, Vodicka E, La Rosa S, Valderrama M, Garcia PJ. Point-of-Care Testing for Anemia, Diabetes and Hypertension: A Pharmacy-Based Model in Lima, Peru. Ann Glob Health. 2017;83(2):394–404. 10.1016/j.aogh.2017.03.514 28619417

[36] Cheema E, Sutcliffe P, Singer DR. The impact of interventions by pharmacists in community pharmacies on control of hypertension: a systematic review and meta-analysis of randomized controlled trials. Br J Clin Pharmacol. 2014;78(6):1238–47. 10.1111/bcp.12452 24966032 PMC4256613

[37] Garcia PJ, Carcamo CP, Garnett GP, Campos PE, Holmes KK. Improved STD syndrome management by a network of clinicians and pharmacy workers in Peru: The PREVEN Network. PLoS One. 2012;7(10):e47750 10.1371/journal.pone.0047750 23082208 PMC3474757

[38] García PJ, Holmes KK, Cárcamo CP, Garnett GP, Hughes JP, Campos PE, et al Prevention of sexually transmitted infections in urban communities (Peru PREVEN): a multicomponent community-randomised controlled trial. Lancet. 2012;379(9821):1120–8. 10.1016/S0140-6736(11)61846-1 22341824 PMC3315635

