# Supplementary material for: Knowledge, attitudes and practices related to tuberculosis in pharmacy workers in a cross-sectional survey in El Agustino, Peru
Source: PLoS One. 2018 Jul 24;13(7):e0196648. doi: 10.1371/journal.pone.0196648 (PMC6057627; doi:10.1371/journal.pone.0196648)
Supplement: S1 File — Survey used to collect data from participants. (PDF) [file pone.0196648.s002.pdf]

Código ____________________ Fecha _____/_____/___201__ Hora_________
mes día año

ENCUESTA SOBRE TUBERCULOSIS Y USO DE TECNOLOGIAS DE LA INFORMACIÓN Y COMUNICACIÓN EN EL AGUSTINO PARA PERSONAL DE FARMACIAS Y BOTICAS

Estimado (a) participante estamos tratando de determinar cuáles son los conocimientos, actitudes y practicas del personal que trabaja en farmacias y boticas con respecto a tuberculosis y uso de tecnologías de la información y comunicación. Esta encuesta es totalmente anónima, los resultados del trabajo se analizarán y se publicarán y esperamos nos permitirán guiar futuras actividades de capacitación. Les agradecemos su participación y respuestas sinceras.

SECCION 1

101. ¿Qué tipo de establecimiento es?
1. ❑ Botica
2. ❑ Farmacia

102.- ¿Pertenece el establecimiento a una cadena de boticas y/o farmacia?
1. ❑ Si
2. ❑ No

103.- Nombre del establecimiento_____________________________

104.- Dirección del establecimiento____

105. Referencia_________________________

106.- ¿Qué días y en que horario atiende?

Día Horario	Desde horas	Hasta horas
106.1. Lunes a Viernes		
106.2. Sábados		
106.3. Domingos		

107.	¿Cuántos turnos al día atiende la botica o farmacia?	______
108.	¿Cuántas horas dura cada turno en promedio?	_____ horas
109.	¿Cuántos trabajadores atienden clientes por turno en promedio?	_____ trabajadores
110.	¿Cuántos trabajadores atienden clientes en el establecimiento en todos los turnos?	______ trabajadores en total
111.	¿Cuál es el número de trabajadores en la botica o farmacia según profesión?	1. Quimico farmacéutico ( )<br>2. Técnico de farmacia ( )<br>3. Otro ( )
112.	¿Cuántos clientes atiende la botica o farmacia durante un turno en promedio?	___ clientes

106. ¿Cuál es el género del encuestado?
1.❑ Mujer 2.❑ Hombre

107. ¿Cuántos años cumplidos tiene? [ ] años

108.- ¿En la farmacia o botica donde trabaja, usted es propietario?
1.❑ No 2.❑ Si

Continúe en la siguiente pagina ➔

116. ¿En la farmacia o botica donde trabaja, usted es …? **[Marque solo una respuesta]**

1. ❑ Químico farmacéutico(a)
2. ❑ Dependiente (sin estudio de farmacia)
3. ❑ Técnico de farmacia

88. ❑ Otro(s) Especifique: ____________________

Piense en un grupo de 10 clientes que ha atendido hoy día o ayer. De esos 10 clientes:

	Número
117. ¿Cuántos preguntaron algo y no compraron nada?	
118. ¿Cuántos compraron algún medicamento con receta?	
119. ¿Cuántos compraron algún medicamento sin receta?	
120. ¿ Cuántos compraron algún producto o servicio no relacionado a salud? ejemplo recarga de crédito para llamadas	
121. ¿Cuántos compraron algún producto o servicio relacionado a salud que no es medicamento? ejemplo pañales	

SECCION 2

201. Marque el tiempo que lleva aproximadamente trabajando en farmacias o boticas en general? (Convertir el tiempo a meses)______ meses

202. Antes de comenzar a trabajar en esta botica o farmacia, ¿Recibió alguna capacitación de la misma botica o farmacia?

1. ❑Si 2. ❑no

203. En los últimos 12 meses, ¿Ha recibido alguna capacitación en algún tema relacionado a salud?

1. ❑Si
2. ❑No – IR A LA 206

204. ¿De quién recibió la capacitación? **Puede marcar más de una opción.**

1. ❑ Empleador/ dueño
2. ❑ Ministerio de salud
3. ❑ Municipio
4. ❑ Universidad
5. ❑ Laboratorio farmacéutico

88. Otra entidad (especifique por favor)_____________

205. ¿De qué tema trató la capacitación? **Puede marcar más de una opción.**

1. ❑ Productos farmacéuticos
2. ❑ Diagnóstico de enfermedades frecuentes
3. ❑ Manejo de enfermedades frecuentes

88. ❑ Otro tema (especifique por favor)_____________

206. ¿Si un cliente con tos productiva (con flema) por más de 15 días viene a la farmacia que le recomienda usted? Sólo debe marcar una alternativa **NO LEER LAS ALTERNATIVAS**

1. ❑ Venderle un producto que le calme la tos
2. ❑ Venderle un antibiótico
3. ❑ Recomendarle que vaya al centro de salud
4. ❑ Decirle que tiene tuberculosis
5. ❑ No sabe

207. ¿Cuál es la molestia o el tema que más le consultan los clientes?
1. ❑ Problemas respiratorios como resfrío, tos
2. ❑ Problemas digestivos como diarrea o dolor abdominal
3. ❑ Problemas de la piel
4. ❑ Dolor de cabeza, músculos o articulaciones
5. ❑ Problemas genitourinarios
88. ❑ Otro problema (especifique por favor)_____________

¿Tiene oportunidad de realizar las siguientes actividades en su trabajo?

208. ¿Tiene oportunidad de conversar con el cliente sobre su molestia o enfermedad?
1.❑ No (IR A 211) 2.❑ Si

209. ¿Si tiene oportunidad de hacerlo que tanto le gusta conversar con los clientes sobre su molestia o enfermedad?
1. ❑ Me gusta mucho
2. ❑ Me gusta poco
3. ❑ Me gusta muy poco
4. ❑ No me gusta

210. ¿Cuántas veces al día puede conversar con los clientes sobre su molestia o enfermedad?
______

211. ¿Qué lo limita para conversar con los clientes sobre sus molestias más frecuentemente? Puede marcar más de una alternativa
1. ❑ Tengo poco tiempo
2. ❑ No me siento capacitado para eso
3. ❑ Necesito pedir permiso a alguien
4. ❑ No tengo limitaciones
5. ❑ Otro problema (especifique por favor)_____________

212. ¿Tiene oportunidad de darle al cliente recomendaciones sobre su tratamiento?
1.❑ No (IR A 215) 2.❑ Si

213. ¿Si tiene oportunidad de hacerlo que tanto le gusta dar recomendaciones de tratamiento?
1. ❑ Me gusta mucho
2. ❑ Me gusta poco
3. ❑ Me gusta muy poco
4. ❑ No me gusta

214. ¿Cuántas veces al día puede dar recomendaciones a los clientes sobre su tratamiento?
______

215. ¿Qué lo limita a dar recomendaciones a los clientes sobre su tratamiento más frecuentemente? Puede marcar más de una alternativa
1. ❑ Tengo poco tiempo
2. ❑ No me siento capacitado para eso
3. ❑ Necesito pedir permiso a alguien
4. ❑ No tengo limitaciones
5. ❑ Otro problema (especifique por favor)_____________

216. ¿Tiene oportunidad de enseñarle al cliente a usar algún producto, ejemplo inhalador?
1.❑ No (IR A 219)   2.❑ Si

217. ¿Si tiene oportunidad de hacerlo que tanto le gusta enseñar a usar un producto?
1. ❑ Me gusta mucho
2. ❑ Me gusta poco
3. ❑ Me gusta muy poco
4. ❑ No me gusta

218. ¿Cuántas veces al día puede enseñarle al cliente a usar un producto?
______

219. ¿Qué lo limita a enseñarle al cliente a usar un producto más frecuentemente?
Puede marcar más de una alternativa
1. ❑ Tengo poco tiempo
2. ❑ No me siento capacitado para eso
3. ❑ Necesito pedir permiso a alguien
4. ❑ No tengo limitaciones
5. ❑ Otro problema (especifique por favor) _____________

220. ¿Con qué frecuencia recomienda al cliente acudir al médico o centro de salud?
1. ❑ Nunca   2. ❑ Pocas veces   3. ❑ Frecuentemente

SECCION 3

301. ¿Ha escuchado hablar acerca de la tuberculosis?
1.❑ No   2.❑ Si

302. ¿Qué tan informado se considera usted respecto a la tuberculosis?
1. ❑ Muy bien informado
2. ❑ Bien informado
3. ❑ Poco informado
4. ❑ Nada informado

303. ¿Cómo se sentiría Ud. al hablar acerca de **tuberculosis** con sus clientes?
1.❑ Muy cómodo (a)   4.❑ Muy incómodo
2.❑ Cómodo   5.❑ No sabe
3.❑ Incómodo

304. ¿Ha tenido o tiene a alguien cercano a Ud. (por ejemplo: familiar, amigo o conocido cercano) que haya enfermado de Tuberculosis?
1.❑ No   2.❑ Si   88.❑ No sabe

305. ¿Es la Tuberculosis una enfermedad contagiosa?
1.❑ No es contagiosa
2.❑ Es poco contagiosa
3.❑ Es muy contagiosa
4.❑ No sé.

306. ¿Cómo se contagia la tuberculosis? Marcar TODO lo que responde **NO LEER LAS ALTERNATIVAS**.

1. ❑ Por darle la mano a un paciente con TBC
2. ❑ Por el aire cuando la persona con TBC tose o estornuda
3. ❑ Al tocar los mismos objetos que toco una persona con TBC
4. ❑ Al usar el mismo baño que una persona con TBC
5. ❑ Por contacto sexual
6. ❑ Por la sangre
7. ❑ Otra (especifique por favor)______
88. ❑ No sabe

307. ¿Cómo puede una persona evitar el contagio de tuberculosis? Marcar TODO lo que responde **NO LEER LAS ALTERNATIVAS.**

1. ❑ Evitar saludar dando la mano
2. ❑ Cubriéndose la boca o la nariz al toser o estornudar
3. ❑ Evitar compartir los cubiertos y platos
4. ❑ Con una buena nutrición
5. ❑ Viajando con las ventanas abiertas en el bus
6. ❑ Otra (especifique por favor) ______
88. ❑ No sabe

308. ¿Todas las personas que tienen TBC tienen síntomas?

1. ❑ Sí.
2. ❑ No.
88. ❑ No sabe.

309.¿Cuál es el síntoma más común de TBC? **NO LEER LAS ALTERNATIVAS**

1. ❑ Fiebre.
2. ❑ Tos.
3. ❑ Estornudos.
4. ❑ Cansancio.
5. ❑ Dolor de pecho.
6. ❑ Pérdida de peso
7. ❑ Otra (especifique por favor) ______
88. ❑ No sabe

310. ¿Cuál es el mejor método para saber si alguien tiene TBC?**LEER LAS ALTERNATIVAS**

1. ❑ Examen de una muestra de esputo (flema)
2. ❑ PPD (inyectar una sustancia en la piel y ver si se forma una pequeña ampolla).
3. ❑ Radiografía (del pecho).
4. ❑ Cultivo de muestra sanguínea.
5. ❑ Otro (especifique por favor) ______
88. ❑ No sabe

311.¿La TBC puede curarse?

1. ❑ Siempre
2. ❑ Casi siempre.
3. ❑ Casi la mitad de las veces
4. ❑ Casi nunca.
5. ❑ Nunca
88. ❑ No sabe

312.¿Qué pasa si una persona con TBC no toma su tratamiento correctamente o toma tratamiento incompleto? Marque todas las alternativas que considere **LEER LAS ALTERNATIVAS**

1. ❑ Hará TBC resistente a medicamentos.
2. ❑ Contagiará a otras personas.
3. ❑ Desarrollará TBC en sitios fuera del pulmón.
4. ❑ Todas las anteriores.

88. ❑ No sabe.

313. ¿Cuál es la mejor forma en que puede curarse una persona con tuberculosis? **LEER LAS ALTERNATIVAS**

1. ❑ Medicina natural, plantas medicinales
2. ❑ Buena nutrición
3. ❑ Combinación de medicamentos que da el centro de salud
4. ❑ Medicinas que se pueden comprar en farmacias
5. ❑ Otra (especifique por favor) ______

88. ❑ No sabe

SECCIÓN 4

Para cada una de las afirmaciones acerca de la *tuberculosis* **en la tabla que se presenta a continuación señale cual es su actitud marcando una de las alternativas siguientes: "Totalmente de acuerdo", "De acuerdo", "En desacuerdo", "Totalmente en desacuerdo", "No se"**

	**1.Totalmente de acuerdo**	**2.De acuerdo**	**3.En desacuerdo**	**4.Totalmente en desacuerdo**	**5. No se**
401. La tuberculosis es frecuente en el Perú	❑	❑	❑	❑	❑
402. La tuberculosis es frecuente en El Agustino	❑	❑	❑	❑	❑
403. La tuberculosis puede causar la muerte	❑	❑	❑	❑	❑
404. Encontrar todos los casos nuevos de tuberculosis es importante para controlarla	❑	❑	❑	❑	❑
405. Es importante involucrar a la comunidad en el control y prevención de la tuberculosis	❑	❑	❑	❑	❑
406. La tuberculosis resistente al tratamiento es un problema grave en el Perú:	❑	❑	❑	❑	❑

407. En la comunidad, ¿cómo nota Ud. que se trata a la gente con Tuberculosis generalmente? **LEER LAS ALTERNATIVAS**

1. ❑ La mayoría de la gente los apoya
2. ❑ La mayoría de la gente la rechaza
3. ❑ La mayoría de la gente la apoya, pero se aleja de ellos
4. ❑ La gente los trata con indiferencia
5. ❑ La gente los trata igual que a los que no tienen TBC

SECCIÓN 5.

501. ¿Le gustaría recibir información sobre la tuberculosis y participar en prevención y control en tu comunidad?

1.❑ No (IR A 503) 2.❑ Si

502. ¿Por qué medio le gustaría recibir información sobre la tuberculosis? **Puede marcar más de una opción**

1. ❑ Charla
2. ❑ volante
3. ❑ Por celular (mensaje de texto)
4. ❑ Por internet (correo electrónico)

88. Otro (especifique por favor)___________________

503. ¿Cuál de los siguientes equipos tiene usted para uso personal? **Puede marcar más de una opción**

1. ❑ celular tipo smartphone
2. ❑ celular que no es smartphone
3. ❑ tablet
4. ❑ laptop
5. ❑ computadora de escritorio

88. ❑ Otro (especifique por favor)___________________

504. ¿Dónde tiene conexión a internet? **Puede marcar más de una opción**

1. ❑ En mi casa
2. ❑ Cabina de internet pública
3. ❑ En la farmacia
4. ❑ En mi celular
5. ❑ No tengo acceso a internet – IR A LA 506

505. ¿Para que utiliza el Internet? **Puede marcar más de una opción**

1. ❑ email
2. ❑ chat
3. ❑ ver videos
4. ❑ juegos
5. ❑ información del trabajo
6. ❑ otro tipo de información
7. ❑ red social (ejemplo Facebook)
8. ❑ Otro (especifique por favor)_____________________

506.- ¿Le gustaría participar en una reunión almuerzo de conversación o grupo focal sobre el tema de Tuberculosis y las farmacias cerca de su establecimiento?

1. ❑ Si
2. ❑ No

507.- número de celular / RPM /RPC ________________________

508.- Correo electrónico __________________________________

MUCHAS GRACIAS POR PARTICIPAR
